# *Streptomyces violaceusniger* WZS5–6 suppresses *Fusarium oxysporum* f. sp. *cubense* tropical race 4 via antifungal metabolites and host defense induction

**DOI:** 10.3389/fpls.2026.1863092

**Published:** 2026-08-19

**Authors:** Wenjing Ge, Tao Jing, Xiaoping Zang, Hafiz Faiq Bakhat, Tianyu Li, Xueyong Zi, Zheli Ding, Mohmed A. M. Abdalhi, Yingdui He, Lixia Wang, Jianghui Xie, Tianyan Yun

**Affiliations:** 1 National Key Laboratory of Tropical Crop Breeding, Institute of Tropical Bioscience and Biotechnology & Sanya Research Institute, Chinese Academy of Tropical Agricultural Sciences (CATAS), Sanya, Hainan, China; 2 Sanya Institute of Henan University, Henan University, Sanya, Hainan, China; 3 Department of Environmental Sciences, COMSATS University Islamabad, Vehari, Pakistan; 4 School of Tropical Agriculture and Forestry, Hainan University, Sanya, China; 5 Department of Agricultural Engineering, Faculty of Agricultural Technology and Fish Sciences, Al-Neelain University, Khartoum, Sudan

**Keywords:** biological control, defense related enzymes, *Fusarium* wilt of banana, metabolomics, *Streptomyces violaceusniger*

## Abstract

**Introduction:**

Fusarium wilt of banana (FWB), caused by *Fusarium oxysporum* f. sp. *cubense* tropical race 4 (Foc TR4), poses a serious threat to the safety and sustainable development of the banana industry. Biological control represents one of the most environmentally friendly approaches for managing this disease.

**Methods:**

In this study, *Streptomyces violaceusniger* WZS5–6 antifungal activity against Foc TR4 has been investigated through an integrated approach combining antifungal assays, genome analysis, and metabolomic profiling. For the purpose, the effects of the bacterial strain and its cell-free extract on morphological and ultrastructural changes on pathogenic fungal hyphae and spores were assessed using scanning and transmission electron microscopy. LC–MS analysis was used to identify the metabolites responsible for antifungal activity. We further explored the potential of *S. violaceusniger* WZS5–6 against Foc TR4 through *in planta* validation.

**Results:**

*Streptomyces violaceusniger* WZS5–6 exhibited a strong inhibition rate of 91.57% on Foc TR4. The cell-free extract obtained from *S. violaceusniger* WZS5–6 strongly inhibited Foc TR4 with an EC_50_ value of 91.62 µg·mL^-1^, indicating the presence of antifungal bioactive metabolites. The results showed that *S. violaceusniger* WZS5–6 significantly inhibited the mycelial growth of Foc TR4 and induced alterations in spore morphology, mycelial ultrastructure, and cell membrane leakage. Metabolomic profiling of the *S. violaceusniger* WZS5–6 extracts revealed numerous antifungal metabolites, among which the key metabolites, viz., citronellic acid and furanodienone, exhibited strong inhibitory effects on Foc TR4, with antifungal activity of 61.13% and 57.44%, respectively. Moreover, strain WZS5–6 not only demonstrated 61.54% control efficacy against FWB in a pot experiment but also showed promising growth-promoting effects on banana plants.

**Discussion:**

This study demonstrates that *S. violaceusniger* WZS5–6 inhibits Foc TR4 through a multi-level mechanism involving cellular disruption, metabolic adaptation, and activation of host defense responses. These findings highlight the potential of *S. violaceusniger* WZS5–6 as a promising novel candidate strain to be employed as a biological control agent of FWB.

## 1 Introduction

Banana (*Musa* spp.) is one of the most popular fruits in tropical and subtropical regions and the fourth largest food crop worldwide (Liu et al., 2025a). It not only has the largest international fresh fruit trade volume but also serves as a staple food for more than 400 million people globally (Kema et al., 2021). However, due to the rapid spread of soil-borne pathogens (*Fusarium oxysporum* f. sp. *cubense*, Foc), the safety and sustainable development of bananas face serious threats. In particular, *Fusarium oxysporum* f. sp. *cubense* tropical race 4 (Foc TR4) poses a serious threat to the healthy development of the global banana industry because of its high incidence rate, destructive potential, and rapid spread (He et al., 2024). Currently, the traditional chemical fungicides used to control FWB proved to be inadequate, with additional economic burden to the growers (Bidabadi and Sijun, 2018). Moreover, the extensive use of chemical fungicides poses a threat to human health and causes environmental pollution. Additionally, the long-term use of chemical fungicides disrupts the balance of soil micro-ecosystem, thereby exacerbating the occurrence of FWB. Given the limited efficacy of chemical control and the scarcity of disease-resistant banana varieties, the adoption of environmentally friendly and sustainable biological control represents a highly promising and relatively effective strategy for managing FWB (Yan et al., 2024; Santos, 2025; Yu et al., 2025).

Biological control using microorganisms has gained scientific attention as a promising strategy for managing soil-borne plant pathogens. *Streptomyces*, with their wide genetic diversity, ease of survival in soil, ability to produce various antagonistic secondary metabolites, and plant-growth-promoting effects, are important biocontrol agents against fungal diseases (Khan et al., 2023). The phytopathogen-suppressing effects of actinomycetes can be attributed to their ability to produce antibiotics, competing for nutrients and space, producing cell-wall-degrading enzymes, and promoting induction/activation of host plant defense responses (Marcos et al., 2025; Santos, 2025). Several studies have reported the antagonistic potential of actinomycetes against the genus *Fusarium*—for instance, *Streptomyces salinarius* Lnu-12 can reduce the abundance of *F. oxysporum* in the rhizosphere and significantly promote pine seedling growth (Wang et al., 2025). *S. hygroscopicus* subsp. *hygroscopicus* 5–4 and its extract exhibited potent antagonistic activity against Foc TR4 through disrupting membrane integrity and ultrastructure (Yun et al., 2023). Similarly, the extracts of *S. yingingensis* sp. nov. YGL11–2 also caused disruption of the Foc TR4 hyphal cell membrane and spore deformation and achieved 41.94% control efficacy against the fungus (Li et al., 2026). Additionally, metabolites from the *Streptomyces* spp. Have shown promising effects against the fungus—for instance, a novel plant resistance inducer, oleamide, isolated from *S. lydicus* JCK-6019, provided 66.28% control against *Fusarium oxysporum* f. sp. *cucumerinum* by maintaining cellular membrane integrity, reduced malondialdehyde accumulation, and enhanced antioxidant defense responses in cucumber (Luo et al., 2025). Similarly, fluvirucin B6 characterized from *S. solisilvae* inhibits Foc TR4 growth by disrupting mitochondrial function and tricarboxylic acid cycle enzyme activities (Chen et al., 2024). Furthermore, integrated untargeted metabolomics (LC–ESI–MS/MS) and genomic analysis identified naringenin, a bioactive flavonoid from *Streptomyces noursei* D337-11, which disrupts cellular integrity by binding to the trypsin and nitroalkane oxidase of Foc TR4 (Zhou et al., 2025). Current research on actinomycetes against Foc TR4 has mainly focused on growth inhibition, spore and hyphal damage, and biocontrol efficacy under plant growth conditions; however, the underlying mechanism remain elusive. Although many studies have shown that extracts of *Streptomyces* are effective against Foc TR4, only a few anti-Foc TR4 metabolites have been reported from this genus to date (Hu et al., 2026).

Microbial interactions trigger the metabolic changes that induce the production of secondary metabolites and also stimulate the production of novel metabolites that are scarce under non-competitive conditions, thereby increasing metabolite diversity (Yun et al., 2025; Emmanuel et al., 2026). Therefore, profiling metabolomic changes during actinomycete–Foc TR4 interactions can provide valuable insights into the dynamic chemical responses underlying biocontrol activity and facilitate the identification of key antifungal metabolites.

In this study, the antifungal activity of *S. violaceusniger* WZS5–6 against Foc TR4 and its potential as a biocontrol agent for FWB were evaluated. For the purpose, the antifungal activity of the strain and its extract were assessed by examining the morphological and ultrastructural changes in fungal hyphae and spores using scanning and transmission electron microscopy. Meanwhile, damage to the Foc TR4 cell membrane and changes in mycelial biomass induced by the strain WZS5–6 extract were evaluated. Integrated genomic sequencing and LC–MS analysis were used to identify the metabolites responsible for antifungal activity. In addition, the *in vitro* antifungal activity of the metabolites was validated in a dual-culture experiment. Finally, the biocontrol efficacy of *S. violaceusniger* WZS5–6 against Foc TR4 was validated under *in vivo* pot experiment with banana plants. Collectively, we hypothesized that *S. violaceusniger* WZS5–6 suppresses Foc TR4 through genome-guided antifungal metabolite production and by inducing host defense responses in banana plants, thereby reducing *Fusarium* wilt incidence and enhancing plant growth. Based on these assumptions, we assume that the findings of this study will provide few novel insights into the potential of *S. violaceusniger* WZS5–6 as a Foc TR4 control agent and elucidate the underlying mechanism of its antifungal activity.

## 2 Materials and methods

### 2.1 Microorganisms and culture conditions

The endophytic actinomycete strain WZS5–6 isolated from the roots of the medicinal plant *Curculigo capitulata* was identified as *Streptomyces violaceusniger* WZS5-6 (GDMCC no. 61984, GenBank ID: JALXFU000000000.1) and maintained on International *Streptomyces* Program 2 (ISP2) at 4 °C (Liu et al., 2025b). Banana wilt disease pathogenic fungi, *Fusarium oxysporum* f. sp. *cubense* tropical race 4 (Foc TR4), was maintained on a potato dextrose agar (PDA) petri dish at 4 °C, and it was provided by the Institute of Tropical Bioscience and Biotechnology, Chinese Academy of Tropical Agricultural Sciences (CATAS), Haikou, China.

### 2.2 Influence of *S. violaceusniger* WZS5–6 on Foc TR4 mycelium development

#### 2.2.1 *In vitro* antifungal activity

The antifungal activity of *S. violaceusniger* WZS5–6 against Foc TR4 was performed using the dual-culture confrontation method (Zhang et al., 2024b). A mycelial disk (5 mm in diameter) of Foc TR4 was placed at the center of a PDA plate, and *S. violaceusniger* WZS5–6 was inoculated at a distance of 2.5 mm from the pathogen. Plates inoculated only with Foc TR4 served as a control. The plates were incubated at 28°C for 7 days. All of the treatments were repeated three times. The inhibition rate was calculated using the following Equation 1:

(1) $$\text{Inhibition rate}=\frac{\text{Diameter of untreated colony}-(\text{diameter of treated colony}-0.5)}{\text{Diameter of untreated colony}}\times 100\%\;$$

In the equation above, 0.5 cm represents the initial fungal mycelial plug diameter used for inoculation that has been subtracted from the treated colony diameter to ensure that the inhibition rate reflects only the newly developed fungal growth.

#### 2.2.2 *In vitro* antagonistic effects of strain WZS5–6 on Foc TR4 mycelial development

The antifungal activity of strain WZS5–6 against Foc TR4 mycelial growth was evaluated using a modified dual-culture method (Yun et al., 2022). A sterile coverslip (1 × 1 cm in size) was placed at the center of a PDA plate, with Foc TR4 and strain WZS5–6 inoculated on opposite ends. After co-culturing at 28°C for 5 days, the coverslip was observed under a microscope (Axio Scope A1, Carl Zeiss AG, Germany).

The influence of strain WZS5–6 on the structure and morphology of Foc TR4 mycelium was observed under a scanning electron microscope (SEM, version S-4800, Hitachi Limited, Japan) and a transmission electron microscope (TEM, version HT7700, Hitachi, Ibaraki, Japan). The samples used for SEM and TEM observations were obtained through the dual-culture confrontation method as described in Section 2.2.1. Inoculation with Foc TR4 was considered the control treatment. Plates co-cultured with Foc TR4 and strain WZS5–6 were incubated at 28 °C for 5 days. The inhibition zone from the plates was excised for SEM and TEM analysis. Samples for SEM observations were fixed, dehydrated, and gold-covered according to the methods reported by Yun et al. (2021). Sample processing for TEM was carried out according to Cao et al. (2022), involving fixation, dehydration, and embedding in Epon 812 resin. Subsequently, the samples were sliced using an ultrathin slicer (Leica, UC6 CM1950, Germany) and stained with 3% (v/v) uranyl acetate and 3% (v/v) lead citrate before the TEM observations. The observations from the SEM and TEM were processed using Adobe Illustrator CS6 (version 16.0, Adobe Systems Incorporated, USA).

### 2.3 Effect of *S. violaceusniger* WZS5–6 extract on Foc TR4 mycelium development

#### 2.3.1 Preparation of extract and EC_50_ determination

The strain, *S. violaceusniger* WZS5-6, was cultured at 28°C for 7 days in soybean liquid medium (SLM) following the method of Liu et al. (2025b) for secondary metabolite production. The fermentation broth was extracted with ethanol (fermentation broth/ethanol = 1:1, v/v), and then the mycelia were removed using a filter paper. The filtrate was concentrated using a rotary vacuum evaporator (R 206D, SENCO, Shanghai, China). The brown residue obtained was stored at 4°C and used for subsequent anti-fungal activity tests.

The antifungal activity of *S. violaceusniger* WZS5–6 extract was determined using agar diffusion method (Ezelarab et al., 2025). The extract was dissolved in sterile water and filtered through a sterile filter membrane with a pore diameter of 0.22 μm. Following a series of dilution methods described by Wang et al. (2021) and Zhang et al. (2022), the extract was added to sterilized PDA medium to prepare drug-containing media with final concentrations of 1.56, 3.12, 6.25, 12.5, 25, 50, 100, and 200 μg·mL^-1^. Each plate was inoculated with Foc TR4 fungus at the center of the plate. The plates were incubated at 28°C for 7 days, and each treatment was repeated thrice. The half-maximal effective concentration (EC_50_) of the extract against Foc TR4 was calculated according to Hoekstra and Van Ewijk (1993).

#### 2.3.2 Effect of the extracts on the dry weight of Foc TR4 mycelium

The pathogenic fungus was inoculated into PDB medium and incubated at 28 °C on a shaker at 180 rpm for 4 days. The spore suspension was filtered and cultured for 3 days on a new PDB medium using 30 µL of the spore suspension. Subsequently, *S. violaceusniger* WZS5–6 extract was added to each group at final concentrations of 1 × EC_50_, 3 × EC_50_, and 6 × EC_50_. After 3 days of incubation, the mycelia were collected by filtration and then processed for dry weight determination. A blank control (sterile water) was included, and each treatment was performed in triplicate.

#### 2.3.3 Effect of the extracts on the cell membrane of Foc TR4 mycelium

An aliquot of Foc TR4 spore suspension (1 mL) was inoculated into 100 mL of PDB medium and cultured at 28 °C with shaking at 180 rpm for 2 days. The extract was added to obtain final concentrations of 1 × EC_50_, 3 × EC_50_, and 6 × EC_50_, with sterile water used as the control group. The spore suspension with extracts were incubated for 3 days and centrifuged at 8,000 *g* for 15 min to obtain the mycelium. The contents of soluble sugar, soluble protein, nuclear leakage, and malondialdehyde (MDA) in Foc TR4 were measured using corresponding detection kits (Beijing Solarbio Science & Technology Co., Ltd., Beijing, China) according to the manufacturer’s instructions. Each assay was performed in triplicate.

#### 2.3.4 Effects of the extracts on the spore morphology and mycelium ultrastructure of Foc TR4

The extract was added to the spore suspension (1.0 × 10^6^ cfu·mL^-1^) to a final concentration of 3 × EC_50_. Sterile water was used as a control. After incubation at 28 °C and 180 rpm for 12 h, a 50-µL aliquot of the sample was placed onto a sterile coverslip (1 × 1 cm) and air-dried naturally. The coverslips were processed according to Section 2.2.2 and observed using a SEM.

To determine the detrimental effects of *S. violaceusniger* WZS5–6 extract on Foc TR4 hyphal cells, the pathogen inoculated to the PDA plates was exposed to an extract concentration of 3 × EC_50_ for 5 days. Samples taken from the colony edge were fixed, dehydrated, and sectioned as described in Section 2.2.2. Changes in the ultrastructure of hyphae cells were observed under a TEM.

### 2.4 Functional gene annotation of *S. violaceusniger* WZS5-6

Based on the previously obtained genome sequence of *S. violaceusniger* WZS5-6 (Liu et al., 2025b), the genome was functionally annotated using the COG (http://www.ncbi.nlm.nih.gov/COG), KEGG (http://www.genome.jp/kegg/),and Carbohydrate-Active Enzymes (CAZy, http://www.cazy.org/) databases. Using the tool of antiSMASH (https://dl.secondarymetabolites.org/releases/4.0.2/), the prediction of secondary metabolite gene clusters in genomes was constructed.

### 2.5 Changes in metabolites from the co-culture of *S. violaceusniger* WZS5–6 and Foc TR4

To understand the changes in the metabolites of *S. violaceusniger* WZS5–6 during co-culture with Foc TR4, the secondary metabolites produced by the strain were identified using LC–MS. Three treatment groups were set up: T1—*S. violaceusniger* WZS5–6 co-culture with Foc TR4 (anti-Foc TR4 activity: 83.65%), T2—*S. violaceusniger* WZS5–6 co-culture with Foc TR4 (anti-Foc TR4 activity: 71.36%), and T3—*S. violaceusniger* WZS5–6 culture alone. Each experiment was performed in triplicate.

The obtained data were processed using the metabolomics software (Progenesis QI) for baseline filtering, peak identification, and integration. The obtained mass spectra were matched against metabolic databases for metabolite annotation. The data matrix was uploaded to the Majorbio Cloud platform (https://www.majorbio.com) for subsequent analyses. After normalization, the standardized data were subjected to principal component analysis (PCA) using the ropls package (Version 1.6.2) in R. Among the treatments, differentially accumulated metabolites (DAMs) were identified based on a variable importance in projection (VIP) score >1, *p*-value <0.05, and an absolute fold change (FC) >1. Finally, a co-expression network was constructed using the WGCNA package, and hub metabolites were identified based on their significant enrichment (*p* < 0.05) within key modules.

### 2.6 Functional validation of key bioactive metabolites by *S. violaceusniger* WZS5-6

The key differential metabolites associated with disease resistance identified by WGCNA were ranked based on their VIP score and Log_2_FC value, with the top 20 metabolites selected. Subsequently, considering factors including compound availability and cost-effectiveness, the corresponding commercial standards were purchased: guanidinoacetic acid, furandienone, citronellic acid, thioacetic acid, 5-fluorouridine, and N-methyltryptamine. Their antifungal activities were evaluated using the agar diffusion assay described in Section 2.3.1. Each treatment was performed in triplicate. The mycelial diameter was measured, and the inhibition rate was calculated.

### 2.7 *In planta* effect of *S. violaceusniger* WZS5–6 on Foc TR4 incidence, plant growth, and host defense response

#### 2.7.1 Banana seedling experiment with pot inoculation

To evaluate the effects of *S. violaceusniger* WZS5–6 on fusarium wilt severity, plant growth, and defense-related enzyme activities in bananas, a pot experiment was performed. The experiment employed a fungus of Foc TR4 expressing green fluorescent protein (GFP). The treatments were designed as follows: (I) control (no Foc TR4, application of water, negative control), (II) Foc TR4 (inoculation with Foc TR4 alone), (III) *S. violaceusniger* WZS5-6 (inoculation with *S. violaceusniger* WZS5–6 alone), and (IV) *S. violaceusniger* WZS5-6 + Foc TR4 (inoculation with both Foc TR4 and *S. violaceusniger* WZS5-6). Banana seedlings (‘Baxi’ banana, AAA) with three to four leaves were transplanted into pots and cultivated in a greenhouse. The disease control effect of *S. violaceusniger* WZS5–6 was determined using the root injury method. The banana seedlings’ roots were treated with 100 mL of Foc TR4 spore suspension (1.0 × 10^6^ cfu·mL^-1^) and *S. violaceusniger* WZS5–6 suspension (1.0 × 10^8^ cfu·mL^-1^), respectively. For each treatment, there were three experimental groups, and each group consists of eight pots.

#### 2.7.2 Evaluation of biological control and growth promotion effects

Colonization of banana roots by green fluorescent protein Foc TR4 was observed using laser scanning confocal microscopy (Olympus Corporation, FV1000, Tokyo, Japan). The collected banana root samples were gently washed with water and sectioned with a blade. Then, intact thin sections were placed on slides for observation under a laser scanning confocal microscope.

The infected banana leaves and bulbs were observed and recorded separately at 45 days post-infection (dpi). The following criteria were used to determine the efficacy of *S. violaceusniger* WZS5–6 against *Fusarium* wilt caused by Foc TR4:

Leaf disease grading: Grade 0 (healthy plants), grade 1 (0%–25% of leaves yellowing), grade 2 (25%–50% of leaves yellowing), grade 3 (51%–75% of leaves yellowing), and grade 4 (76%–100% of leaves yellowing). The disease index (DI) and control effect of *S. violaceusniger* WZS5–6 against Foc TR4 were calculated by using Equations 2, 3 as proposed by Fan et al. (2021).

(2) $$\text{DI }(\%)=\sum \frac{\text{Number of diseased plants at each grade}\times \text{value of relative disease grade}}{\text{Total number of plants}\times \text{maximum disease grade}}\times 100$$

(3) $$\text{Control effect }(\%)=\frac{\text{DI of control plants}-\text{DI of treatment plants}}{\text{DI of control plants }}\times 100$$

The growth parameters of banana seedlings in each treatment group were measured, including plant height, stem diameter, leaf thickness, number of leaves, and plant fresh and dry weights (Mohsen et al., 2025; Shalu et al., 2025).

#### 2.7.3 Measurements of defense-related enzyme activities in banana roots

To assess the physiological responses of banana roots to Foc TR4 infection and the protective effects of *S. violaceusniger* WZS5–6 extracts, enzymatic activities were assessed. After 45 days, the collected banana root samples were cleaned to remove the soil and then washed with sterile water. The samples were wrapped in tin foil, rapidly frozen in liquid nitrogen, and stored at -80 °C. The activities of phenylalanine ammonia-lyase (PAL), polyphenol oxidase (PPO), peroxidase (POD), and catalase (CAT) were measured using assay kits (Beijing Solebao Technology Inc., Beijing, China). Enzyme activities were expressed as units per gram of tissues (U·g^-1^). All of the experiments were performed in triplicate.

### 2.8 Data statistics and analyses

The obtained data set was subjected to analysis of variance (ANOVA) using SPSS version 27 (SPSS Inc., Chicago, IL, USA). Duncan’s multiple-range test was applied to assess statistical significance (*p* < 0.05) between different treatments. All experiments were performed in triplicate, and the results were presented as mean ± standard deviation.

## 3 Results

### 3.1 Influence of *S. violaceusniger* WZS5–6 on Foc TR4 mycelium development

#### 3.1.1 *In vitro* antifungal activity of *S. violaceusniger* WZS5–6 against Foc TR4

**Figure 1:**
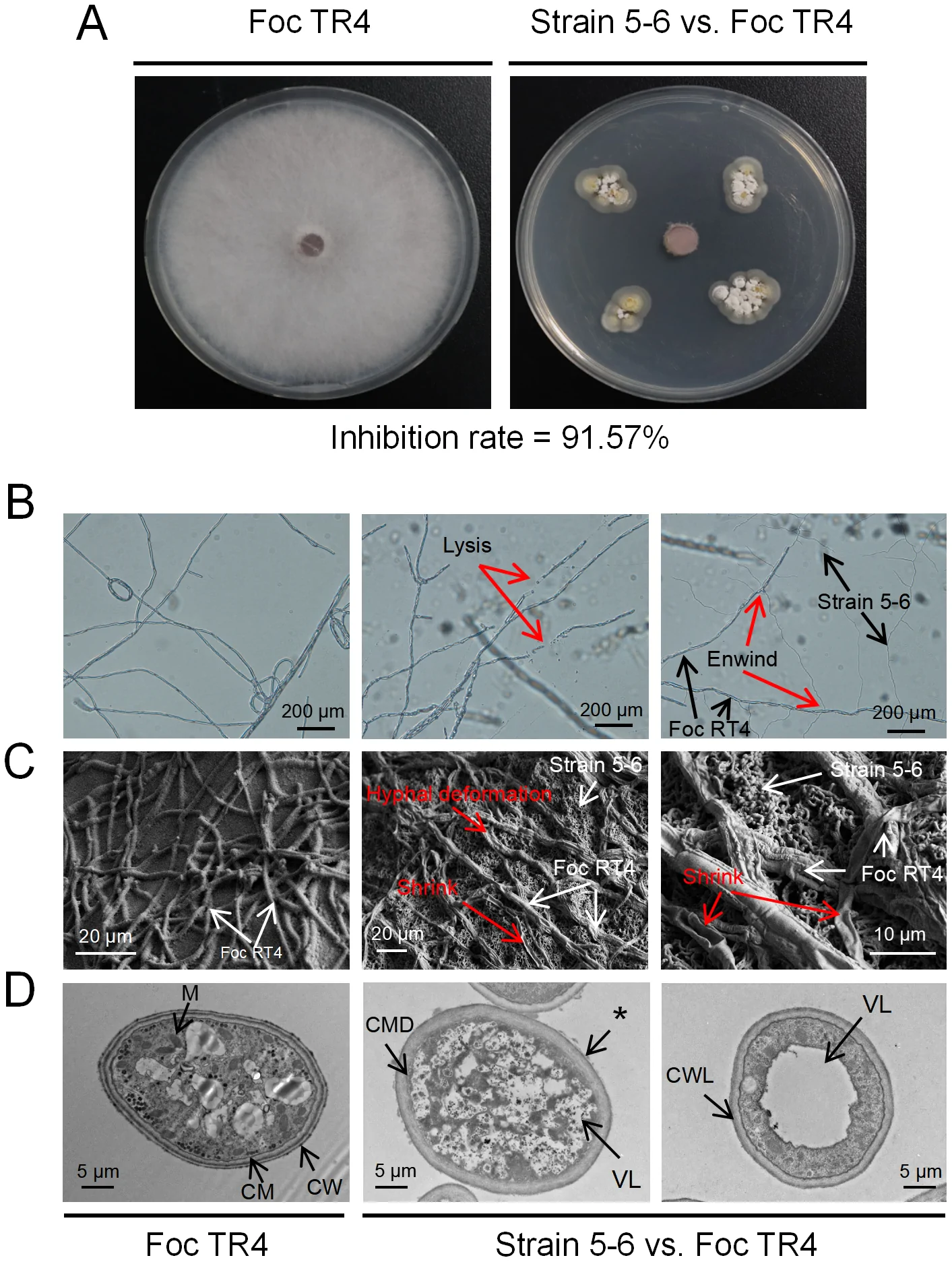
Effect of *S. violaceusniger* WZS5–6 on the mycelial development and ultrastructure of Foc TR4. **(A)** *S. violaceusniger* WZS5–6 with a high antifungal activity against Foc TR4. **(B)** Inhibitory effects of *S. violaceusniger* WZS5–6 on the growth of Foc TR4 mycelial as observed using a light microscope. **(C)** SEM observation of the inhibitory effect of *S. violaceusniger* WZS5–6 on the mycelial morphology of Foc TR4. **(D)** Effect of *S. violaceusniger* WZS5–6 on the ultrastructural of mycelium as observed using TEM. M, mitochondria; *thickened and irregular cell walls; VL, vacuolization; CWL, cell wall lysis; CMD, cell membrane damage.

Strain WZS5–6 showed a strong antifungal activity against Foc TR4, with an inhibition rate of 91.57% (**Figure 1A**). A clear inhibition zone in dual-culture plates was observable at the interaction zone, indicating strong antagonistic activity.

#### 3.1.2 Effects of *S. violaceusniger* WZS5–6 on Foc TR4 mycelial morphology

The inhibitory effects of *S. violaceusniger* WZS5–6 on the mycelial growth of Foc TR4 was observed using light microscope, SEM, and TEM. In the dual-culture experiment, *S. violaceusniger* WZS5–6 and Foc TR4 exhibited an intertwined growth, and Foc TR4 hyphae showed signs of lysis under light microscope (**Figure 1B**). After 5 days of co-culture, the SEM observations revealed significant structural changes in Foc TR4 hyphae, including mycelial shrinkage and deformation (**Figure 1C**). In contrast, the untreated Foc TR4 hyphae was linear and smooth.

#### 3.1.3 Effect of *S. violaceusniger* WZS5–6 on the mycelial ultrastructure of Foc TR4 *in vitro*

To investigate the co-culturing effects of *S. violaceusniger* WZS5–6 on the ultrastructural changes in Foc TR4, hyphae were examined using TEM. In the untreated mycelia, intact fungal structures such as the cell wall (CW), cell membrane (CM), mitochondria (M), and vesicles (V) appeared normal and well-organized (**Figure 1D**). The mycelium treated with *S. violaceusniger* WZS5–6 showed reduced and condensed intracellular matrix, while the cytoplasmic contents became disorganized. The cell wall of Foc TR4 mycelium exhibited irregular thickening, formation of multi-layered structure, and degradation. The cytoplasm was reduced, concentrated, and clearly vacuolated. Intracellular organelles were difficult to distinguish, with most of them damaged and undergoing hydrolysis.

### 3.2 Influence of extract on Foc TR4 mycelium development

#### 3.2.1 Antifungal activity and EC_50_ of *S. violaceusniger* WZS5–6 extracts against Foc TR4

Extracts at different concentrations exhibited an inhibitory effect on Foc TR4 mycelial growth in a dose-dependent manner. The inhibition rates of Foc TR4 were 12.66%, 16.62%, 23.22%, 21.37%, 25.07%, 33.51%, 48.28%, and 54.09% at *S. violaceusniger* WZS5–6 cell-free extract concentrations of 1.56, 3.12, 6.25, 12.50, 25, 50, 100, and 200 µg·mL^-1^, respectively (**Supplementary Figure 1**), although a slight fluctuation in Foc TR4 inhibition was observed between 6.25 and 12.50 µg·mL^-^¹. However, this variation falls within the experimental error of the triplicate assays, while the overall inhibition trend remained concentration dependent. The EC_50_ of the extract *S. violaceusniger* WZS5–6 against Foc TR4 mycelial growth was 91.62 μg·mL^-1^.

**Figure 2:**
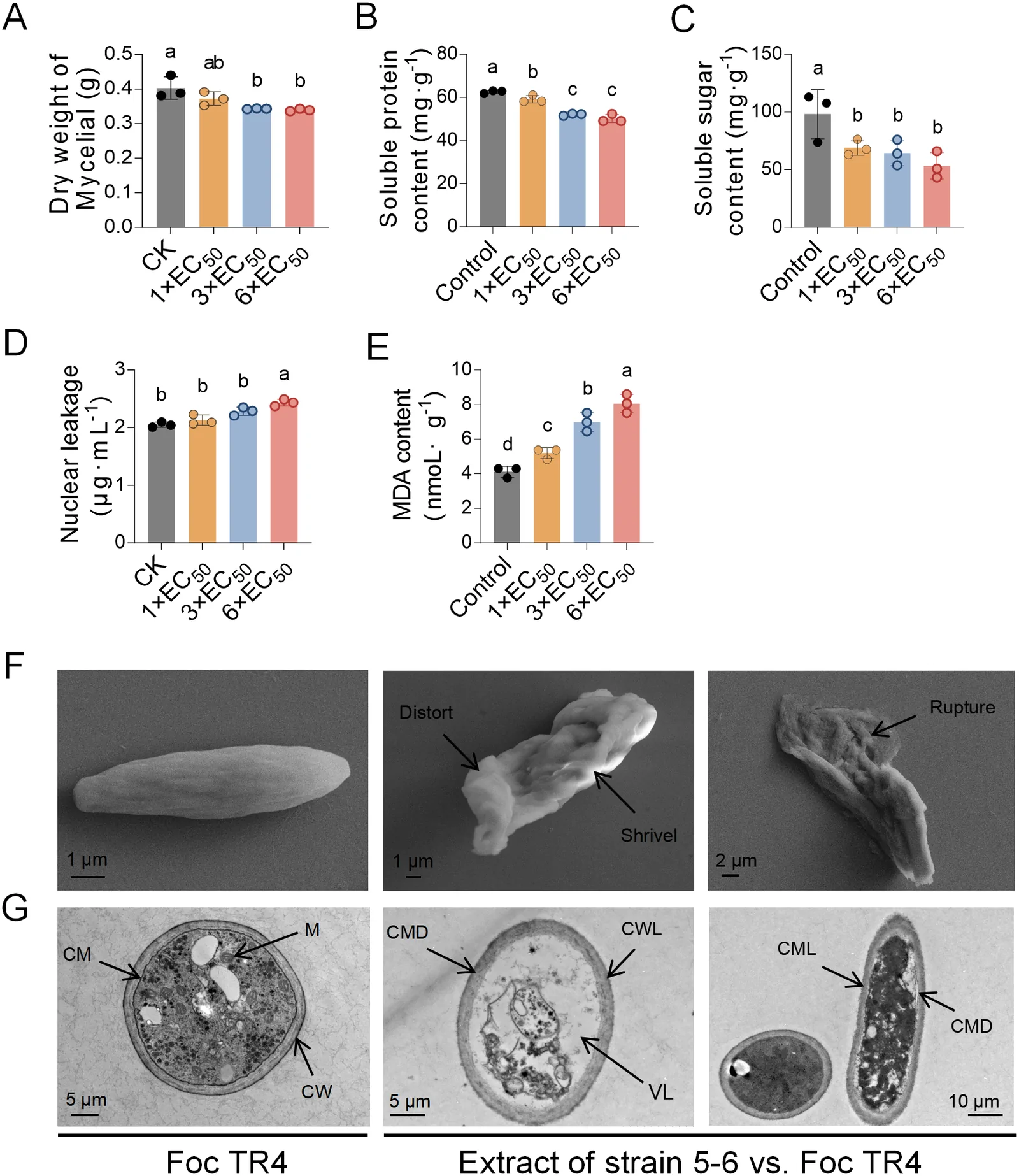
Effects of *S. violaceusniger* WZS5–6 extract on the mycelial and spore morphology and cell membrane integrity of Foc TR4. **(A)** Effect of extracts from *S. violaceusniger* WZS5–6 on the dry weight of Foc TR4 mycelial. **(B–E)** The cell membrane integrity of Foc TR4 after treatment with the extract is characterized by the contents of soluble protein (**B**), soluble sugar **(C)**, nuclear leakage **(D)**, and MDA **(E, F)** Effects of extracts on Foc TR4 spore morphology as observed using SEM. **(G)** Effects of extracts on the ultrastructure of mycelium as observed using TEM. M, mitochondria; *thickened and irregular cell walls; VL, vacuolization; CWL, cell wall lysis; CMD, cell membrane damage; control: Foc TR4 treated with sterile water; 1 × EC_50_, Foc TR4 treated with 91.62 μg·mL^-1^ extract; 3 × EC_50_, Foc TR4 treated with 274.86 μg·mL^-1^ extract; 6 × EC_50_, Foc TR4 treated with 549.72 μg·mL^-1^ extract. Different lowercase letters indicate significant differences (*p* ≤ 0.05).

As the concentration of the extract increased, the dry weights of Foc TR4 mycelia gradually decreased (**Figure 2A**). The mycelia dry weight of the 6 × EC_50_, 3 × EC_50_, and 1 × EC_50_ treatment groups were 0.339, 0.343, and 0.372 g, respectively, and were significantly lower than that of the control group (0.403 g). These results confirm the inhibitory effect of *S. violaceusniger* WZS5–6 against Foc TR4 mycelium growth. There was no significant difference in mycelial dry weight between the 6 × EC_50_ and 3 × EC_50_ groups.

#### 3.2.2 Effect of *S. violaceusniger* WZS5–6 extracts on the cell membrane of Foc TR4

To investigate the physiological mechanism underlying the inhibitory effect of *S. violaceusniger* WZS5–6 cell-free extract against Foc TR4, the membrane integrity of the fungal mycelium was determined by measuring the contents of soluble sugar, soluble protein, nuclear leakage, and MDA. After exposure to the extract, the nuclear leakage and MDA content of Foc TR4 was increased, whereas the contents of soluble sugars and soluble proteins were decreased (**Figures 2B–E**).

The soluble protein contents were significantly higher in the control compared to the cell-free-extract-treated Foc TR4 groups. Foc TR4 treated with the extract at concentrations of 1 × EC_50_, 3 × EC_50_, and 6 × EC_50_ showed 5.50%, 16.86%, and 19.87% less soluble protein contents, respectively, compared to the control treatment (**Figure 2B**). Similarly, the soluble sugar contents in the control group were highest (98.26 mg·g^-1^) in the control treatment. Foc TR4 treated with *S. violaceusniger* WZS5–6 at the concentrations of 1 × EC_50_, 3 × EC_50_, and 6 × EC_50_ showed 29.65%, 34.25%, and 45.62% less soluble sugar contents, respectively, than that of the control group. The soluble sugar contents decreased as the concentration of the extract increased (**Figure 2C**).

As shown in **Figure 2D**, the extract of *S. violaceusniger* WZS5–6 induced a dose-dependent increase in nucleic acid leakage from Foc TR4 mycelia. Compared with the control group, the absorbance at 260 nm was increased in all of the treated groups. The nucleic acid leakage of 1 × EC_50_, 3 × EC_50_, and 6 × EC_50_ groups were 3.90%, 11.35%, and 18.66% higher, respectively, than that of the control group.

Damage to the cell membrane results in the release of MDA. The results show that Foc TR4 exposure to cell-free extracts caused membrane disintegration, with an increased concentration of MDA (**Figure 2E**). The MDA contents in the 1 × EC_50,_ 3 × EC_50_, and 6 × EC_50_ treatment groups were 26.21%, 69.66%, and 95.71% higher, respectively, than that of the control group.

The effects of the 3 × EC_50_ and 6 × EC_50_ groups on the cell membrane of Foc TR4 were comparable. Therefore, the extract with a concentration of 3 × EC_50_ was selected for the subsequent experiments.

#### 3.2.3 Extract induced spore morphological, mycelial ultrastructural, and membrane damage to Foc TR4

To further understand the antifungal mechanism of *S. violaceusniger* WZS5-6, we examined the effect of 3 × EC_50_ extract on the spore and mycelial morphology of Foc TR4 using SEM and TEM. The spores in the control group were intact and plump. In contrast, the spores treated with 3 × EC_50_ extract led to severe morphological abnormalities and structural damages, including distortion, rupture, and lysis. This indicates a loss of spore cellular integrity in Foc TR4 after treatment with the *S. violaceusniger* WZS5–6 extract (**Figure 2F**).

The TEM observations revealed ultrastructural changes in Foc TR4 hyphae following exposure to the 3 × EC_50_ extract. In the control group, the hyphal cell walls, membranes, mitochondria, and vesicles were intact and visible (**Figure 2G**). In contrast, treatment with the *S. violaceusniger* WZS5–6 extract resulted in disorganization, disorientation, vacuolation, blurred matrix, and organelle lysis in Foc TR4 cells. Notably, the integrity of Foc TR4 cell wall and cell membrane was destroyed. Consistent with these disturbances in the cell membrane, the results also showed that *S. violaceusniger* WZS5–6 extract significantly damaged the hyphal ultrastructure integrity. The cell wall of Foc TR4 mycelia undergoes degradation, with the outer wall showing fibrous digestion.

### 3.3 Genome analysis using bioinformatics

**Figure 3:**
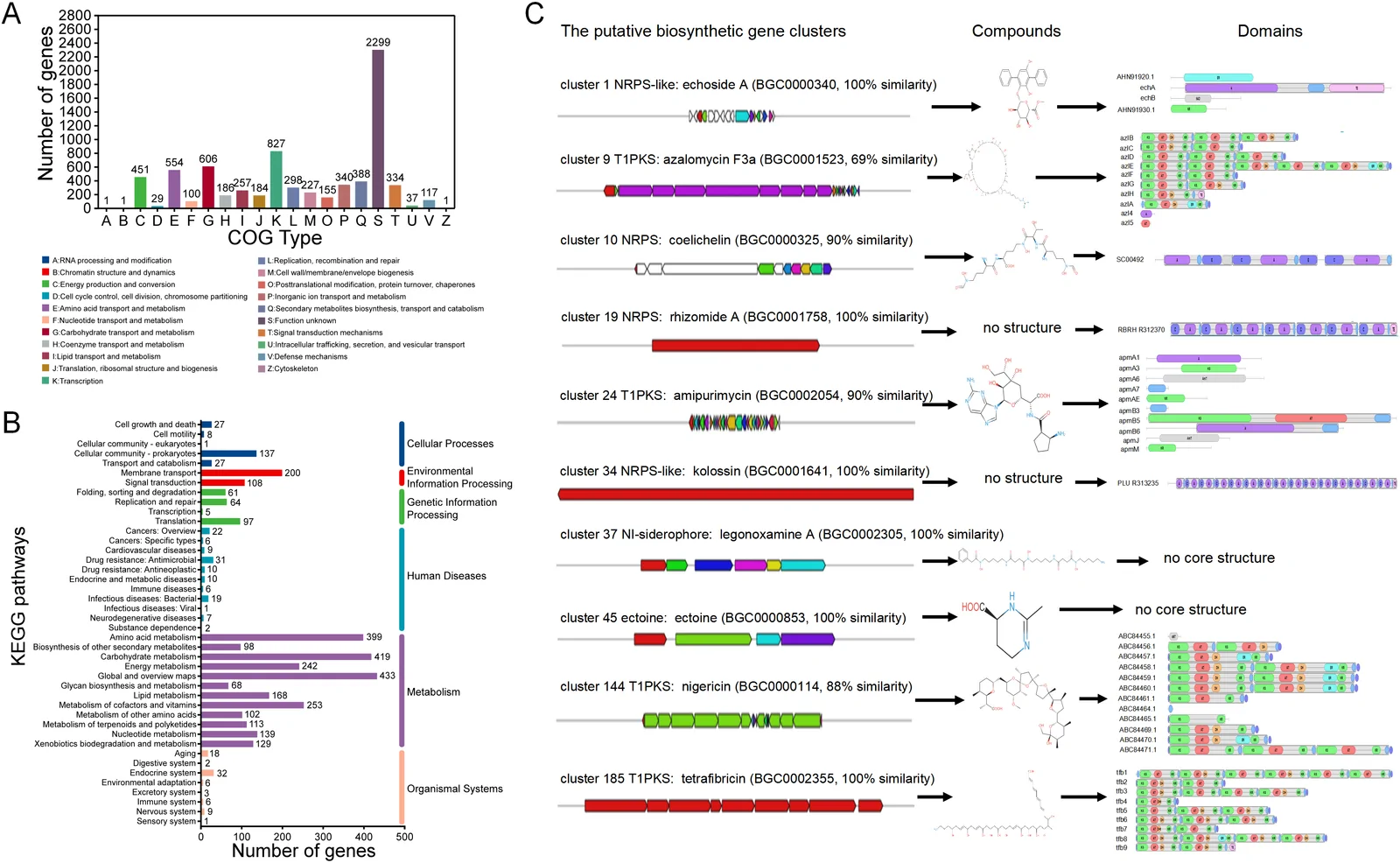
Functional annotation of *S. violaceusniger* WZS5–6 genes. **(A)** COG functional classification. **(B)** KEGG classification of metabolic pathways. **(C)** Biosynthetic gene clusters and core structures were predicted using AntiSMASH. The core structures of 10 BCGs owned more than 69% of similarity with the known BCGs.

Actinomycetes are well known for producing antibiotic metabolites, especially under natural conditions. Genomic sequencing was employed to explore the genetic potential of this strain, particularly regarding secondary metabolite biosynthesis. According to bioinformatics analysis, 7,272 genes were successfully annotated by COG. Among these, 388 genes (5.34%) were associated with secondary metabolite biosynthesis, transport, and catabolism, while 117 genes (1.61%) were related to defense mechanisms (**Figure 3A**). Based on the KEGG annotation, 42 pathways including 3,225 genes were closely associated with the biosynthesis of metabolites. Specifically, 113 genes were involved in the metabolism of terpenoids and polyketides, while 98 genes were related to the biosynthesis of other secondary metabolites, 168 genes were involved in lipid metabolism, and 68 genes were involved in glycan biosynthesis and metabolism (**Figure 3B**). Notably, a large number of unidentified functional genes may be involved in the regulation of the biosynthesis of secondary metabolites.

A total of 63 biosynthetic gene clusters (BGCs) of secondary metabolites were predicted by comparative analysis of genome sequence. These BGCs were responsible for the production of antimicrobial metabolites such as nigericin, amipurimycin, desferrioxamin B, echoside A, and azalomycin F3a. The core structures of nine BGCs exhibited more than 70% similarity to known BGCs (**Figure 3C**). Cluster 1 responsible for producing antimicrobial echoside A exhibited 100% similarity to *Streptomyces* sp. LZ35 (Zhu et al., 2014). Meanwhile, cluster 24 displayed 90% similarity to the BGC of amipurimycin from *S. novoguineensis* (Kang et al., 2019). Cluster 144 showed 88% similarity with the BGC of nigericin from *S. violaceusniger* (Harvey et al., 2007). The core structures of clusters 19, 34, 37, 45, and 185 were found to exhibit 100% similarity with the BGCs of rhizomide A, kolossin, legonoxamine A, ectoine, and tetrafibricin, respectively.

In addition, the genome of *S. violaceusniger* WZS5–6 was compared with the Carbohydrate Active Enzymes database (CAZy, http://www.cazy.org/) using the Diamond and Hmmscan software. *S. violaceusniger* WZS5–6 has the capability to produce 424 carbohydrate-active enzymes (CAZymes), including chitinases (GH18), chitosanases (GH75), β-(1,3)-glucanases (GH55), and xyloglucanase (GH16) (**Supplementary Figure 2**). This finding is consistent with our previous report that *S. violaceusniger* 5–6 produces cell-wall-degrading enzymes (Liu et al., 2025b), and it is further supported by TEM observations showing the disruption of the Foc TR4 cell wall (**Figure 1D** and **2G**).

### 3.4 Changes in metabolites from the co-culture of *S. violaceusniger* WZS5–6 and Foc TR4

#### 3.4.1 Metabolome data quality and differentially accumulated metabolite identification

**Figure 4:**
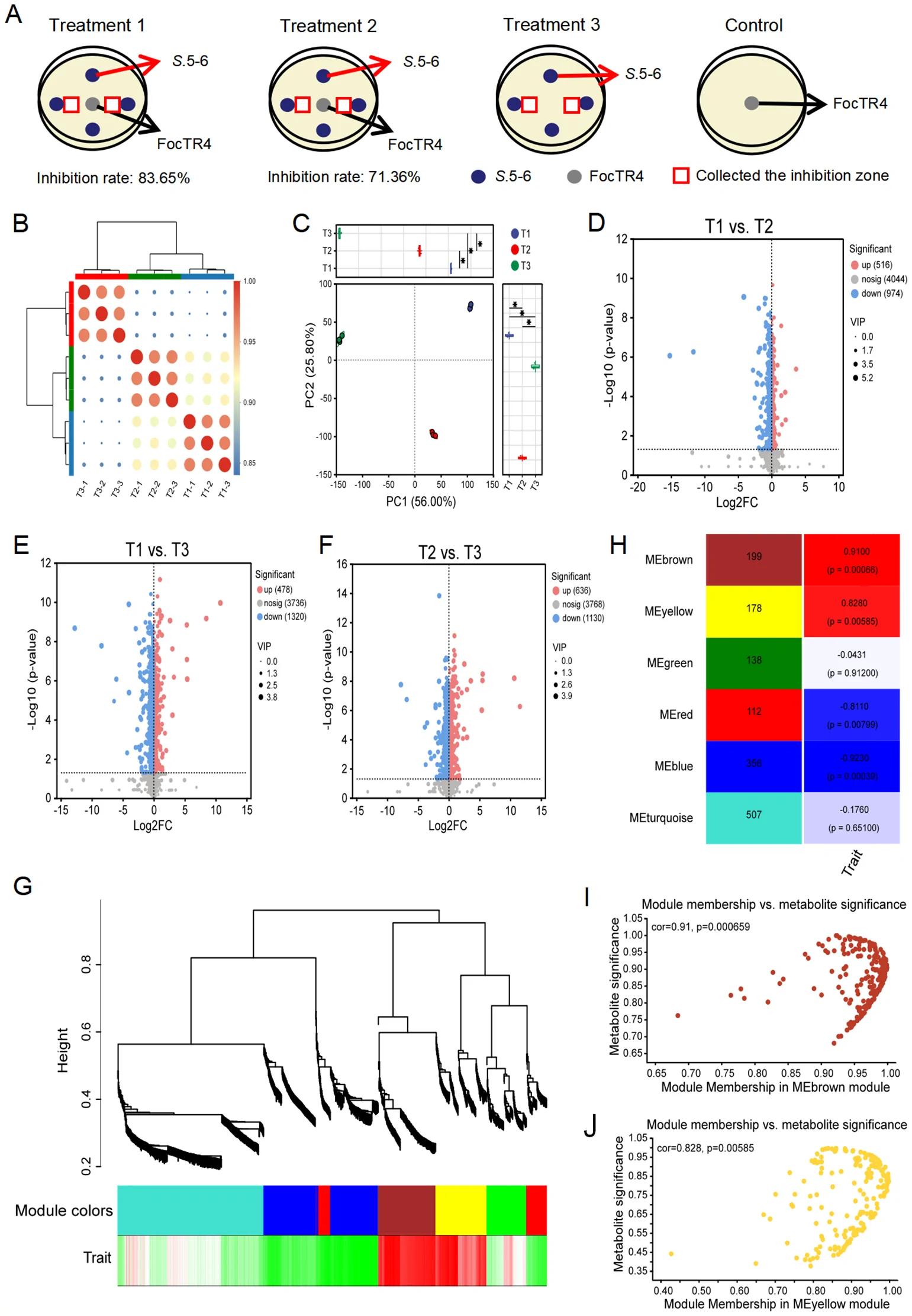
Identification of the key DAMs in *S. violaceusniger* WZS5–6 during co-culture with Foc TR4. **(A)** Dual-culture assay of *S. violaceusniger* WZS5–6 with Foc TR4. Dual-culture setup: After co-culturing the mycelia on PDA medium for 5 days, the surface mycelia were gently scraped for metabolomic analysis. **(B)** Pearson correlation analysis: Different colors represent the relative magnitude of correlation coefficients between samples. **(C)** PCA analysis: A longer inter-sample distance suggests that the samples are more distinct from each other. **(D–F)** Volcano plot: Each point corresponds to a metabolite; point size encodes the VIP score. Red point indicates significantly upregulated, blue point indicates significantly downregulated, and gray point indicates non-significant metabolites. **(G)** Identification of modules by co-expression analysis. A total of six distinct modules were identified. **(H)** Heatmap of module–trait correlations. The numbers on the left indicate the number of metabolites in different modules, and the data on the right show the correlation coefficients and significance (*p*-values) between the modules and the trait. **(I, J)** MM–GS scatter plot of MEbrown module **(I)** and MEyellow module (**J**). By calculating the MM value (i.e., kME) for each metabolite within the module, a MM–GS scatter plot is obtained.

Non-targeted metabolic changes during the co-culture of *S. violaceusniger* WZS5–6 and Foc TR4 were monitored using LC–MS (**Figure 4A**). Based on the Pearson correlation coefficients of metabolite levels between different samples, a strong correlation (0.9252) between samples T1 and T2 was observed. In comparison, the correlations for T1–T3 and T2–T3 were 0.8531 and 0.8619, respectively (**Figure 4B**). The principal component analysis (PCA) revealed distinct metabolic separations among the treatment groups (**Figure 4C**). PC1 and PC2 explained 56.00% and 25.80% of the variance, respectively. The differentially accumulated metabolites (DAMs) were screened with the criteria of VIP >1, *p* < 0.05, and |FC| >1. In T1 vs. T2, a total of 516 upregulated and 974 downregulated DAMs were identified (**Figure 4D**). For T1 vs. T3, 478 DAMs were upregulated and 1,320 were downregulated (**Figure 4E**). Similarly, T2 vs. T3 revealed 636 upregulated and 1,130 downregulated DAMs (**Figure 4F**).

#### 3.4.2 Identification of the key disease-resistant DAMs by WGCNA

A weighted gene co-expression network analysis (WGCNA) was performed on the 1,490 DAMs identified from T1 vs. T2 (**Supplementary Table 1**). A phenotype data vector was constructed, in which antifungal activity was binary-coded (0 = good antifungal activity, 1 = moderate antifungal activity or no antifungal activity) to represent the resistance trait across samples (**Supplementary Table 2**). To identify the key metabolite modules associated with the antifungal activity of *S. violaceusniger* WZS5-6, the correlation coefficients between traits and each metabolite modules were calculated. Based on the metabolite–phenotype correlation heatmap, the metabolites were co-clustered into six modules (**Figure 4G**). The MEred and MEblue modules were significantly negatively correlated with the trait. The differential modules consisted of 468 DAMs: MEred (112 DAMs, correlation coefficient = -0.8110, *p* = 0.00799) and MEblue (356 DAMs, correlation coefficient = -0.923, *p* = 0.00039) (**Figure 4H**). Most notably, the MEbrown and MEyellow modules demonstrated a significant positive correlation with the trait. The differential modules consisted of 377 DAMs: MEbrown (199 DAMs, correlation coefficient = 0.9100, *p* = 0.00066) (**Figure 4I**) and MEyellow (178 DAMs, correlation coefficient = 0.8280, *p* = 0.00585) (**Figure 4J** and **Supplementary Table 4**), whereas there were no differences (*p* > 0.05) among the other modules. The differential modules of MEbrown and MEyellow (contained 377 DAMs) were selected for subsequent analysis.

#### 3.4.3 Screening and validating the antimicrobial activity of DAMs

**Figure 5:**
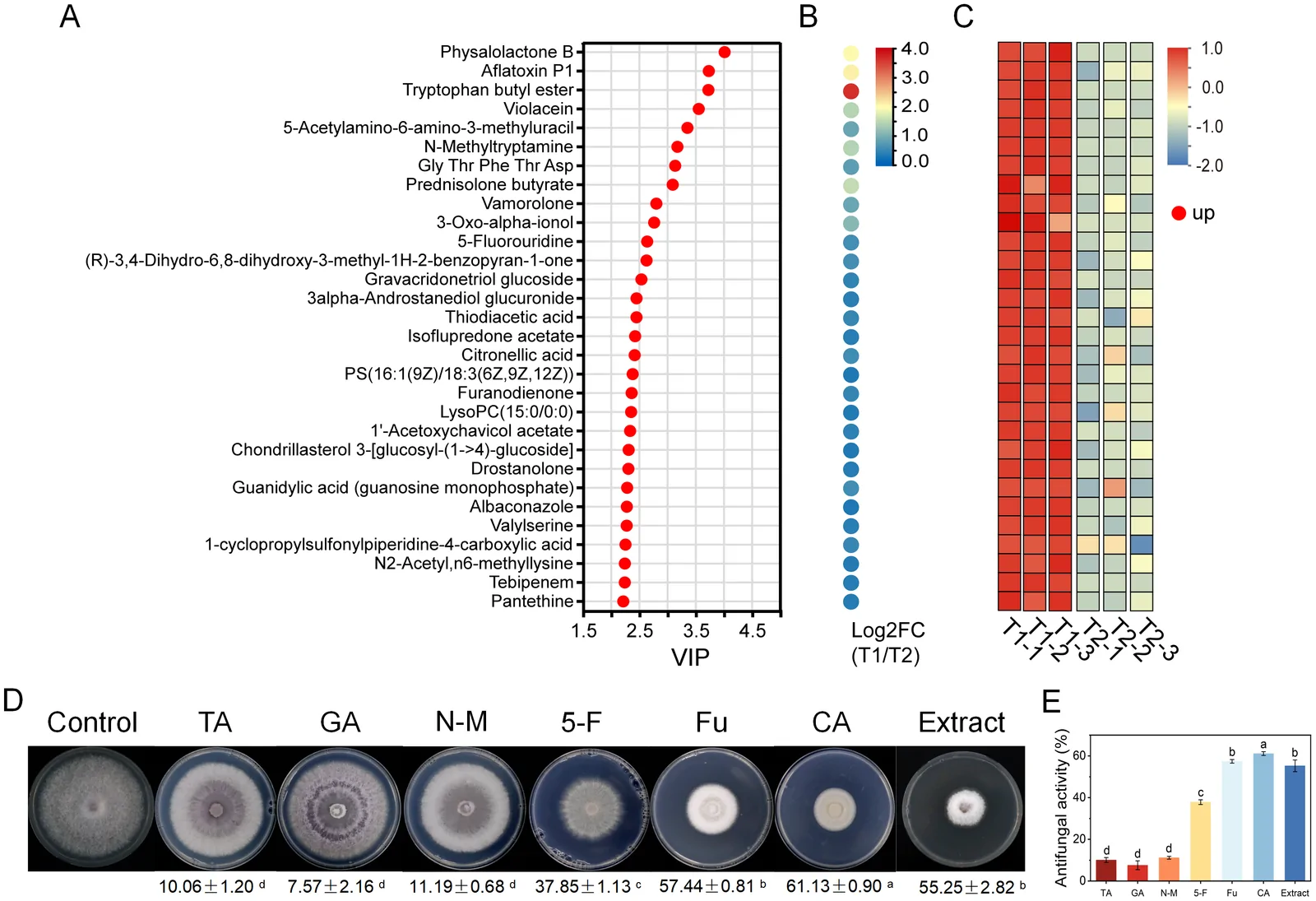
Screening and validating the antimicrobial activity of DAMs. **(A)** VIP scores. The top 30 metabolites of the MEbrown and MEyellow modules were sorted according to their VIP values. The VIP score was based on the PLS-DA model. The selected metabolites were those with VIP >2. **(B)** Log_2_FC of T1 and T2 groups. **(C)** Abundance ratio of heat map. The heat map with red boxes indicates a high abundance ratio of the corresponding metabolite in T1 and T2 groups. **(D–E)** Validating the antimicrobial activity. Antifungal activities at a concentration of 100 μg/mL against Foc TR4. Different lowercase letters indicate significant differences (*p* < 0.05).

Based on the weighted coefficients of the PLS-DA model, the top 30 metabolites in the MEbrown and MEyellow modules were ranked according to their variable importance in projection (VIP) score (**Figure 5A**). The VIP values of metabolites were all greater than 2. The following metabolites especially exhibited the highest VIP scores: physalolactone B (VIP = 4.01), aflatoxin P1 (VIP = 3.73), tryptophan butyl ester (VIP = 3.72), violacein (VIP = 3.55), 5-acetylamino-6-amino-3-methyluracil (VIP = 3.35), N-methyltryptamine (VIP = 3.17), Gly Thr Phe Thr Asp (VIP = 3.13), prednisolone butyrate (VIP = 3.09), 5-fluorouridine (VIP = 2.64), thiodiacetic acid (VIP = 2.45), citronellic acid (VIP = 2.42), furanodienone (VIP = 2.36), and guanidylic acid (VIP = 2.28). All identified metabolites exhibited Log_2_FC values above 0.3 in T1 vs. T2 as follows: tryptophan butyl ester (3.65), aflatoxin P1 (2.09), physalolactone B (2.02), prednisolone butyrate (1.54), violacein (1.44), N-methyltryptamine (1.43), 5-fluorouridine (0.58), guanidylic acid (0.57), citronellic acid (0.57), thiodiacetic acid (0.49), and furanodienone (0.48) (**Figure 5B**). The expression levels of the metabolites in the T1 group were higher than those of the T2 group (**Figure 5C**). Although metabolite annotation was based on LC–MS spectral matching with public databases, the identification of various metabolites such as aflatoxin P1 should be considered putative as these were not confirmed using authentic standards.

Considering the VIP score, Log_2_FC value, availability, and cost-effectiveness of metabolites, guanidylic acid, furanodienone, citronellic acid, thiodiacetic acid, 5-fluorouridine, and N-methyltryptamine were selected for antifungal activity evaluation against Foc TR4. Among the tested metabolites, citronellic acid and furanodienone showed strong antifungal activity, with inhibition rates of 61.13% ± 0.90% and 57.44% ± 0.81%, respectively, which were comparable to that of the extract (55.25% ± 2.82%) (**Figures 5D,E**). These results indicate that citronellic acid and furanodienone were mainly associated with the antifungal activity of *S. violaceusniger* WZS5-6, and these metabolites can be tested in applied research for plant disease management.

### 3.5 *In planta* effect of *S. violaceusniger* WZS5–6 on Foc TR4, banana growth, and defense-related enzymes

#### 3.5.1 Effect of *S. violaceusniger* WZS5–6 on the biological prevention of FWB

**Figure 6:**
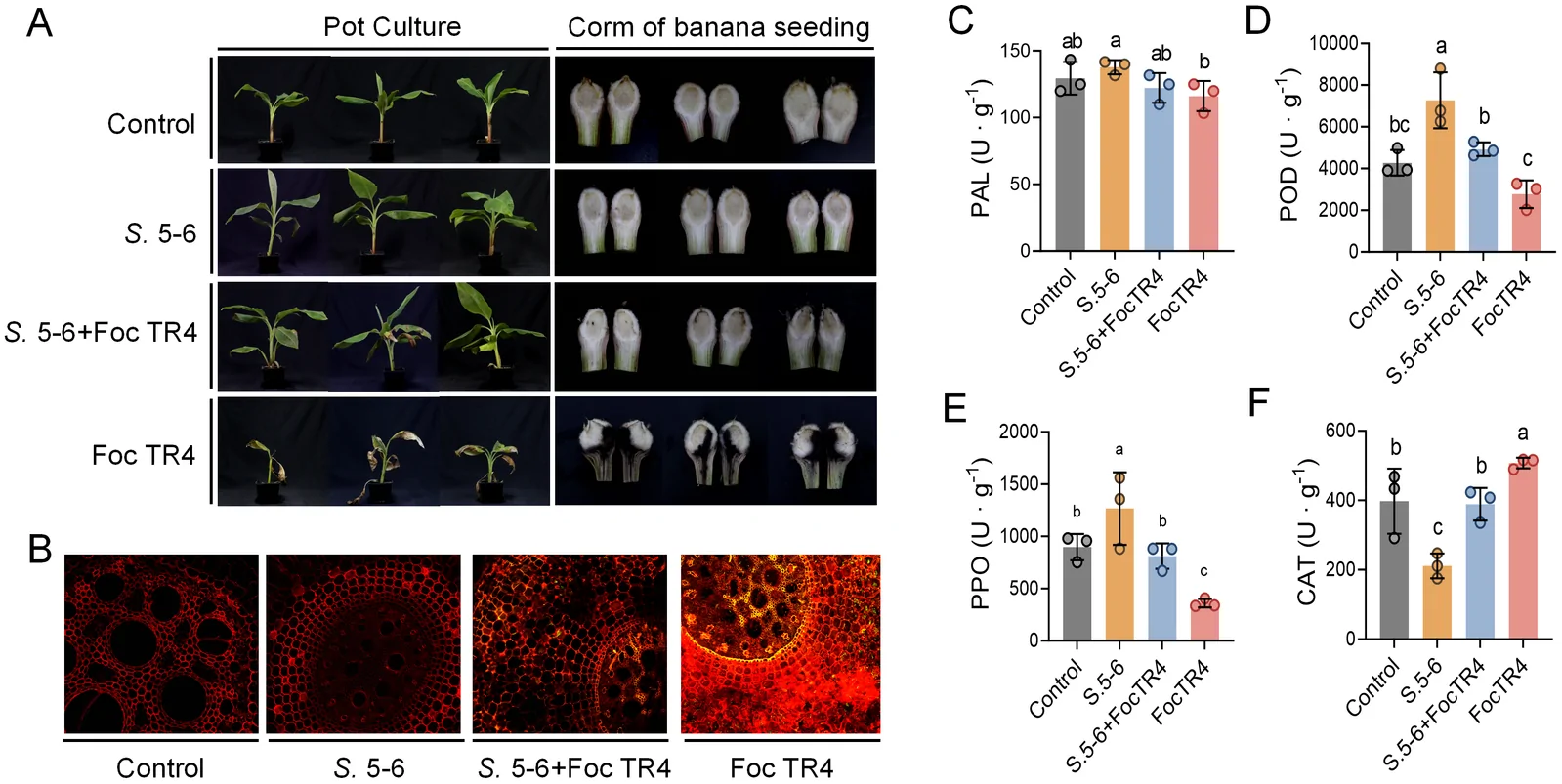
Effects of *S. violaceusniger* WZS5–6 on the control of Foc TR4 and defense-related responses in banana. **(A)** Efficacy of strain WZS5–6 for controlling banana wilt disease. **(B)** Colonization of GFP-Foc4 in banana tissues under different groups. **(C–F)** Enzymatic activity of defense-related enzymes in banana roots: phenylalanine ammonia-lyase (PAL, **(C)**), peroxidase (POD, **(D)**), polyphenol oxidase (PPO, **(E)**), and catalase (CAT, **(F)**). In the graphics, the mean (bars) and the standard error (error bars) are reported. Different letters indicate significant differences at *p ≤*0.05.

At 45 dpi, the banana leaves in the Foc TR4 group exhibited severe wilting and necrosis, with a disease index of 72.22% (**Figure 6A**). In contrast, most banana leaves in the *S. violaceusniger* WZS5-6 + Foc TR4 group remained green and healthy, with a disease index of 27.78%. The banana leaves in the control and *S. violaceusniger* WZS5–6 alone groups remained green and showed no yellowing symptoms. Longitudinal sectioning of banana corm from the Foc TR4 group exhibited widespread black discoloration, indicating severe infection. In contrast, the *S. violaceusniger* WZS5-6 + Foc TR4 group showed only minimal symptoms. Treatment with *S. violaceusniger* WZS5–6 significantly reduced the Foc TR4 infection, with a control effect of 61.54% (**Table 1**).

**Table 1:** Control effect of *S. violaceusniger* WZS5–6 on FWB.

Treatment	Foc TR4	*S. violaceusniger* WZS5-6 + Foc TR4
Disease index (%)	72.22	27.78
Control effect (%)	-	61.54

Each treatment consisted of eight banana seedlings. All experiments were repeated in triplicate..

#### 3.5.2 Inhibitory effect of *S. violaceusniger* WZS5–6 on the colonization of GFP-Foc TR4

Using a laser confocal scanning microscope, the colonization status of GFP-Foc TR4 in banana roots was observed (**Figure 6B**). No green fluorescence was detected in the control and *S. violaceusniger* WZS5–6 groups. In the Foc TR4 group, a large number of GFP-Foc TR4 were observable in the roots. In contrast, only a small amount of GFP-Foc TR4 hyphae and conidia were found attached to the banana roots in the *S. violaceusniger* WZS5-6 + Foc TR4 group, indicating that *S. violaceusniger* WZS5–6 exerted certain inhibitory effects on the infection of GFP-Foc TR4 in the banana root system.

#### 3.5.3 Effects of *S. violaceusniger* WZS5–6 on the activity of plant-defense-related activities in banana roots

Treatment with *S. violaceusniger* WZS5–6 enhanced the internal defense responses of banana plants against the pathogenic fungus Foc TR4. The results showed that inoculation with Foc TR4 suppressed the activities of PAL, POD, and PPO in banana roots, while treatment with *S. violaceusniger* WZS5–6 increased these enzymes’ activities.

The PAL activities in the control, *S. violaceusnige* WZS5-6, *S. violaceusnige* WZS5-6 + Foc TR4, and Foc TR4 groups were 129.43, 137.76, 122.21, and 116.10 U·g^-1^, respectively (**Figure 6C**). Similarly, banana inoculation with Foc TR4 resulted in decreased POD activity, which was alleviated in the presence of *S. violaceusnige* WZS5-6. Among the groups, the highest POD activity was observed in the *S. violaceusnige* WZS5–6 group (7,271.60 U·g^-1^), which was significantly higher than those of the other groups (**Figure 6D**). The POD activity in the *S. violaceusnige* WZS5-6 + Foc TR4 group was 4,922.87 U·g^-1^, which was 1.78-fold higher than that of the Foc TR4 group (2,770.13 U·g^-1^). The Foc TR4 group resulted in significantly lower PPO activity (359.16 U·g^-1)^ in banana roots compared with the control group (897.00 U·g^-1^). The PPO activities in the *S. violaceusnige* WZS5–6 and *S. violaceusnige* WZS5-6 + Foc TR4 groups were 1,266.83 and 810.77 U·g^-1^, respectively, which were significantly higher than that of the Foc TR4 group, representing 3.53- and 2.26-fold increase in enzyme activity, respectively (**Figure 6E**).

In contrast to the above-mentioned enzymes, the CAT activity in banana roots significantly increased in the Foc TR4 group at 45 dpi, while the *S. violaceusnige* WZS5–6 group significantly decreased it. The CAT activity in the Foc TR4 group was 507.48 U·g^-1^, which was 1.28, 2.40, and 1.31-fold higher than those of the control, *S. violaceusnige* WZS5-6, and *S. violaceusnige* WZS5-6 + Foc TR4 groups, respectively (**Figure 6F**).

#### 3.5.4 Plant-growth-promoting effects

**Figure 7:**
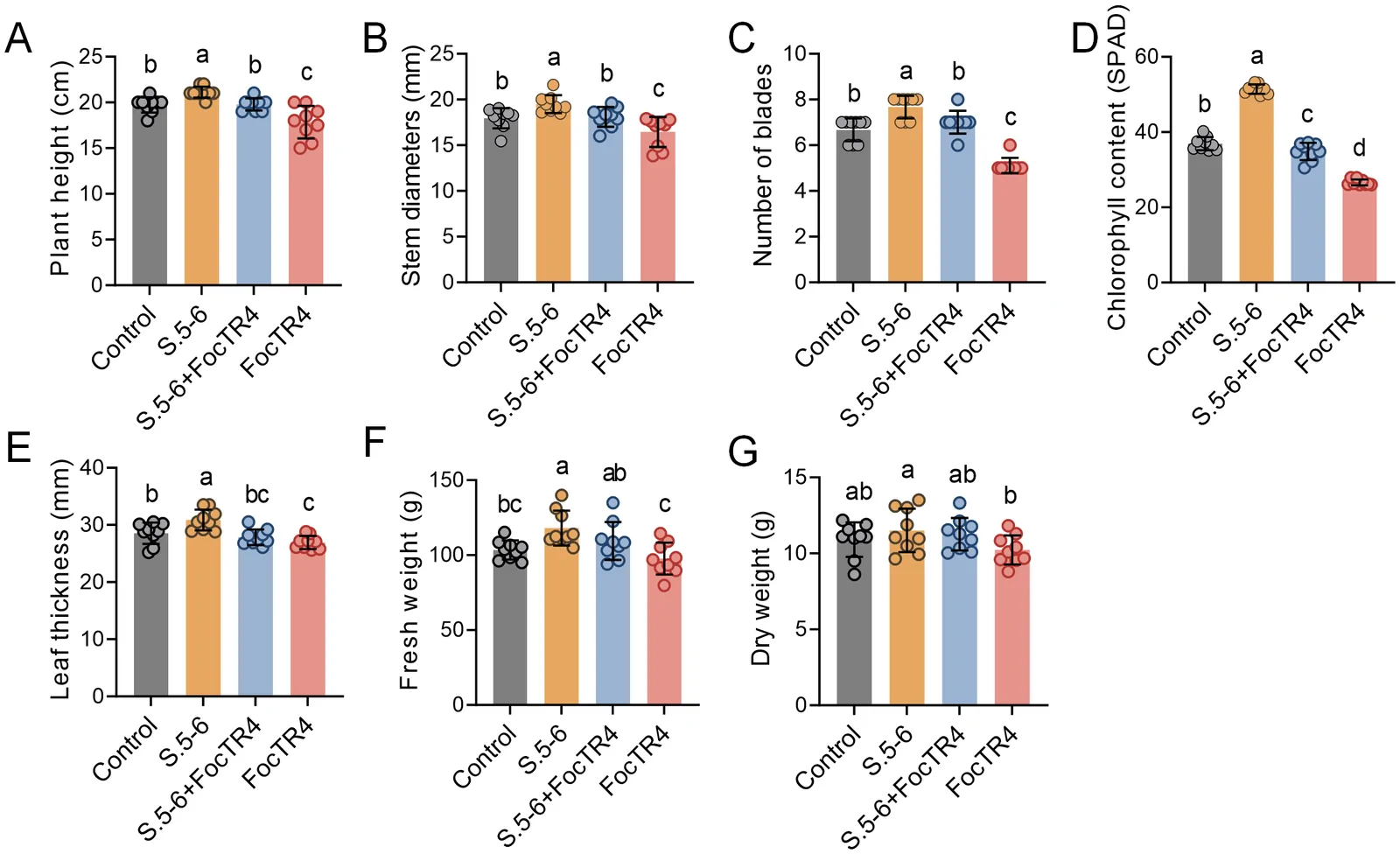
Effects of *S. violaceusniger* WZS5–6 on the growth of banana seedlings in a pot experiment. Growth indexes in the control and treatments of banana: **(A)** plant height, **(B)** stem thickness, **(C)** leaf number, **(D)** leaf thickness, **(E)** chlorophyll content, **(F)** fresh weight, and **(G)** dry weight, measured after 45 dpi. Control group: noninoculated Foc TR4 and application of water, negative control; S.5–6 group: noninoculated Foc TR4 and application of *S. violaceusniger* 5-6; S.5-6 + Foc TR4 group: inoculated Foc TR4 and *S. violaceusniger* WZS5-6; Foc TR4: inoculated Foc TR4, positive control. Different letters indicate significant differences at *p ≤*0.05.

Banana seedlings treated with *S. violaceusniger* WZS5–6 exhibited better growth performance compared to the Foc TR4 and control groups. Plant growth attributes, including plant height, stem diameter, chlorophyll content, number of leaves, and leaf thickness, were significantly changed with the inoculation of Foc TR4 and *S. violaceusniger* WZS5-6 (**Figure 7**). Compared with the Foc TR4 group, inoculation with *S. violaceusniger* WZS5–6 resulted in 18.38% increase in plant height (**Figure 7A**), 18.47% in stem diameter (**Figure 7B**), 50.00% in leaf number (**Figure 7C**), 14.65% in leaf thickness (**Figure 7D**), 93.12% in chlorophyll content (**Figure 7E**), 21% in fresh weight (**Figure 7F**), and 13% in dry weight (**Figure 7G**). Notably, the combination treatment of *S. violaceusniger* WZS5-6 + Foc TR4 showed significantly improved banana growth compared with the Foc TR4 alone group, albeit to a lesser extent than the *S. violaceusniger* WZS5–6 group. Therefore, *S. violaceusniger* WZS5–6 did not only decrease the *Fusarium* incidence caused by Foc TR4 in banana but had also shown promising plant-growth-promoting effects on banana seedlings.

## 4 Discussion

Banana is one of the most widely cultivated and consumed fruits in the world, serving as both a staple food and an important cash crop (Muhammad et al., 2025). However, banana plants are susceptible to disease, especially the *Fusarium* wilt caused by the fungus Foc TR4, which can lead to significant yield losses (Rebouças et al., 2021). The lack of effective fungicides and the long persistence of the pathogen in soil have driven the search for sustainable alternatives, among which biocontrol using antagonistic microorganisms has emerged as a promising strategy (Ruan et al., 2024). In this study, we identified *S. violaceusniger* WZS5–6 as a biocontrol agent with potent activity against Foc TR4. The strain exhibited multiple mechanisms of action, including direct antifungal effects through the disruption of pathogen cellular integrity, production of key secondary metabolites, induction of plant defense responses, and plant growth promotion. Collectively, these findings position the strain as a highly promising candidate for the integrated management of *Fusarium* wilt in banana.

Although previous studies have reported that *S. violaceusniger* produces antibiotics against fungal pathogens (Trejo-Estrada et al., 1998; Sarwar et al., 2019), its specific mechanisms of action against Foc TR4 remain elusive. To investigate the antifungal activity and mechanism of strain WZS5-6, its direct antagonistic activity against Foc TR4 was confirmed through microscopic observation and biochemical analyses. Scanning and transmission electron microscopy revealed that treatment with strain WZS5–6 or its extracts disrupted spore morphology and mycelial ultrastructure, including mycelial deformation, organelle degradation, and loss of cytoplasmic integrity. These results are consistent with the mode of action observed for other biocontrol extracts, where disruption of membrane and organelle integrity leads to fungal growth inhibition (Li et al., 2021). Cell wall degradation of Foc TR4 by enzymes including chitinase and β-1,3-glucanase is one of the main contributory mechanisms by which the *Streptomyces* species can inhibit the activity of Foc TR4. In addition to cell wall and morphological alterations, increased cell membrane permeability as evidenced by the leakage of cytoplasmic and nucleic acid contents was also observed. Damages to Foc TR4 mycelia were evident from the increased MDA content and decreased soluble sugar and soluble protein contents, further supporting the hypothesis that the integrity of the fungal membrane was compromised. Disruption of the plasma membrane compromised the overall cell functioning, leading to the loss of osmolyte and metabolites, which may trigger a cascade of processes that ultimately result in non-functional Foc TR4 hyphae. Such pleiotropic effects on the pathogen suggest that strain WZS5–6 exerts its antifungal action through multiple cellular targets, a feature that is particularly valuable for mitigating the risk of resistance development.

Biocontrol microorganisms and their metabolites play a critical role in establishing stable, disease-suppressive soil microbial communities, thereby reducing the incidence of *Fusarium* wilt in banana. However, the identification of novel bioactive metabolites remains a major challenge (Zhou et al., 2025; Shi et al., 2026). Genomic analyses indicate that *Streptomyces* species possess substantial untapped biosynthetic capacity: most genomes contain 36–48 BGCs, only a minority of which have been linked to known metabolites (Zdouc et al., 2023; Nguyen et al., 2026). Recent advances in genome mining and BGC-activity prediction have revitalized natural product discovery by enabling the targeted exploration of phylogenetically distinct strains and silent metabolic pathways (Nguyen et al., 2026). Genome mining of strain WZS5–6 predicted 63 BGCs, including those encoding polyketide synthases (PKS) and non-ribosomal peptide synthetases (NRPS), which are known to direct the production of diverse bioactive secondary metabolites (Margaret et al., 2024; Wang et al., 2026). A large number of unidentified functional genes may also be involved in regulating their biosynthesis. In addition, genome analysis revealed that strain WZS5–6 has the potential to produce chitinases (*GH18*), chitosanases (*GH75*), β-(1,3)-glucanases (*GH55*), and xyloglucanase (*GH16*). Chitin and β-(1,3)-glucan constitute the major structural components of the fungal cell wall, which functions as an essential barrier for maintaining cell viability and protection. *Trichoderma* deploys an arsenal of potent chitinases to dismantle the cell walls of prey fungi (Deng et al., 2026). Our previous study also confirmed that this strain produces chitinase and β-1,3-glucanase during cultivation (Liu et al., 2025b). The TEM observations further supported these findings, showing the degradation of the Foc TR4 cell wall, indicating that the extracts of strain WZS5–6 cause damage to the cell wall of Foc TR4.

Recent advances in metabolomics have shown that microbial confrontation induces the activation of silent biosynthetic pathways, leading to the production of secondary metabolites with antimicrobial activities. Metabolomic analysis revealed significant changes in the metabolites of strain WZS5–6 during confrontation culture. Six key differential metabolites—guanidylic acid, furanodienone, citronellic acid, thiodiacetic acid, 5-fluorouridine, and N-methyltryptamine—were tested for antifungal activity against Foc TR4. Among these, citronellic acid and furanodienone exhibited antifungal activity comparable to the strain WZS5–6 extract. Furanodienone is a furan sesquiterpene compound with anti-fungal activity against plant pathogenic fungi, making it a promising candidate to be tested as a natural fungicide (Alfie et al., 2017). The compound showed a dose-dependent antifungal activity against *Fusarium solani sensu lato* (Jesmin et al., 2019). Similarly, germacrene D and nootkatone belonging to sesquiterpenes produced by *Streptomyces* (KHY26^T^) suppressed Foc TR4 growth and reduces microconidia germination (Chen et al., 2026). Citronellic acid is a monoterpenoid organic acid. Although there are no direct studies on the antifungal properties of citronellic acid, its related compounds such as citronellol and citronellal have exhibited strong antifungal activity (Zhang et al., 2024a; He et al., 2026). Citronellol-exposed hyphae of *C. camelliae* became thinner, broken, folded, and deformed, with symptoms of inward collapse and separation of the cell wall, as well as degradation of organelles (Zhang et al., 2024a). Although other metabolites displayed lower inhibitory effects than the *S. violaceusniger* extract, their antifungal activity confirms their contribution to pathogen suppression in a potential synergistic way in the presence of multiple metabolites.

However, the metabolites detected through LC–MS analysis, including citronellic acid and furanodienone, could not be directly assigned to specific predicted BGCs. This phenomenon may be attributed to the following two factors: (i) The genome of strain WZS5–6 contains a large number of unidentified functional genes that may be involved in the regulation of secondary metabolite biosynthesis. This condition likely reflects the current limits of genome-mining annotation and database-based cluster recognition (Nguyen et al., 2026). (ii) During co-culture with Foc TR4, the pathogen may activate the biosynthetic regulatory mechanisms of other unidentified secondary metabolites in strain WZS5-6, thereby altering its metabolite profile. Therefore, the inability to assign citronellic acid or furanodienone directly to predicted BGCs is quite probable because *Streptomyces* metabolomics often yields only partial correspondence between predicted clusters and detected compounds (Marta et al., 2025), which shows that genomic presence does not always guarantee the detectable production of the metabolites. As reported in Vignolle et al. (2025) and Tocino-Márquez et al. (2026), *Streptomyces* contains several uncharacterized BGCs that may specify the biosynthesis of novel secondary metabolites. Microbial confrontation induces the activation of silent biosynthetic pathways, leading to the production of secondary metabolites with antimicrobial activities. In addition, the identification of citronellic acid and furanodienone was mainly based on high-resolution mass spectrometry, and their structural and genetic validation still requires further investigation. Therefore, targeted qRT-PCR analysis on key genes involved in the biosynthesis of cell-wall-degrading enzymes, terpenoids, and polyketides in strain WZS5-6, aiming to strengthen the mechanistic link between genomic potential and phenotypic observations, provides an important direction for future studies on the regulatory activation of BGCs in the strain during fungal interactions.

Beyond direct antagonism, the efficacy of biocontrol agents often depends on their ability to prime plant defenses, a phenomenon known as induced systemic resistance (ISR) (Aizada et al., 2026). In the present study, banana plant inoculated with strain WZS5–6 showed a significant reduction in the incidence of FWB which may be associated with ISR-mediated plant defense responses. *Streptomyces* species are known to trigger ISR through signaling pathways involving jasmonic acid and ethylene (Le et al., 2021). The combined effect of direct antifungal activity and ISR resulted in a control efficacy of 61.54% against Foc TR4 in pot experiments. These observations further confirm that the spore disruption and mycelial growth inhibition induced by strain WZS5–6 may have reduced the ability of Foc TR4 in the soil to penetrate and infect banana plants (Palmieri et al., 2020). This level of efficacy is comparable or even higher than many previously reported *Streptomyces* strains used against *Fusarium* wilt in banana or other crops (Getha et al., 2005; Li et al., 2021). Strain inoculation significantly increased the activities of key defense-related enzymes in banana roots, including POD, PPO, and PAL. Consistent with these results, Yun et al. (2022) observed that *Streptomyces* sp. 5–4 activated banana defense mechanisms by upregulating PAL, POD, PR-1, β-1,3-glucanase, LYK-1, and MPK-1. These enzymes are critical for lignification, phytoalexin synthesis, and reactive oxygen species scavenging, all of which contribute to enhanced resistance against fungal invasion (Kaur et al., 2022; Zhang et al., 2022; Qi et al., 2026). The elevation of defense enzyme activities indicates that strain WZS5–6 activates ISR in banana, thereby fortifying the host’s innate immune system.

Moreover, strain WZS5–6 also exhibited plant-growth-promoting effects, as evidenced by increased plant height and biomass in treated plants. Various mechanisms including phytohormones production, enhanced nutrient availability, and suppression of disease-causing microbes may have collectively contributed to increased plant growth. Similar observations have been reported by Li et al. (2021), who found that banana seedlings treated with *Streptomyces* sp. H4 broth showed significantly improved chlorophyll contents, stem diameter, leaf area, plant height, leaf thickness, and root and shoot biomass compared with the control. Overall, the growth-promoting effect of strain WZS5–6 aligns with broader evidence that *Streptomyces* enhances plant growth by improving nutrient availability, stress tolerance, and plant health.

Taken together, this study demonstrates that *S. violaceusniger* WZS5–6 inhibits Foc TR4 through a multi-level mechanism involving cellular disruption, metabolic adaptation, and activation of host defense responses. This provides a field-scale validation of *S. violaceusniger* WZS5–6 as a sustainable biocontrol agent.

## 5 Conclusion

**Figure 8:**
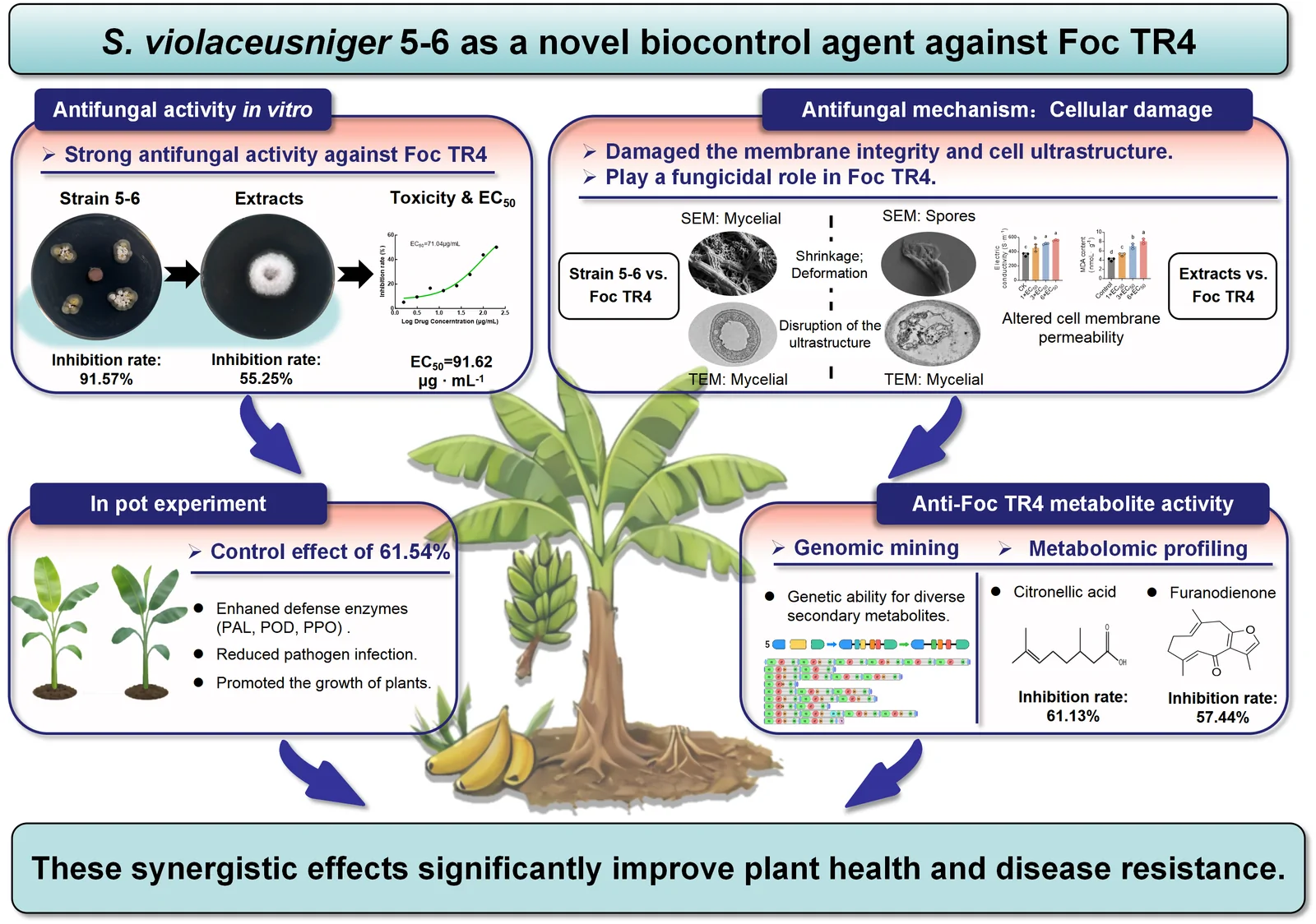
Conceptual model of the biocontrol mechanism of *S. violaceusniger* WZS5–6 against FWB.

*Streptomyces violaceusniger* WZS5–6 demonstrates significant potential as a novel biocontrol agent against Foc TR4 (**Figure 8**). Strain WZS5–6 and its extracts inhibited Foc TR4 growth by disrupting spore morphology, mycelial ultrastructure, and cell membrane integrity. Genomic mining and metabolomic profiling indicated the genetic capacity of *S. violaceusniger* to produce diverse types of secondary metabolites. Among the identified metabolites, citronellic acid and furanodienone were associated with antifungal activity and may be responsible for the suppression of FWB. Furthermore, the pot experiment indicated that *S. violaceusniger* WZS5–6 inoculation reduced pathogen infection and improved banana growth, which were associated with the increased activity of defense-related enzymes. Collectively, these synergistic effects significantly improve the overall plant health and disease resistance. Therefore, *S. violaceusniger* WZS5–6 could be considered as a promising candidate for the sustainable management of FWB. Further investigations are needed to explore and comprehend the potential of *S. violaceusniger* WZS5–6 as a biocontrol agent under field conditions, strengthen the mechanistic link between genomic potential and phenotypic observations via qRT-PCR, and further characterize the mechanisms of the identified bioactive metabolites.

## Data Availability

The datasets presented in this study can be found in online repositories. The names of the repository/repositories and accession number(s) can be found in the article/**Supplementary Material**.
