# Supplementary material for: Streptomyces violaceusniger WZS5–6 suppresses Fusarium oxysporum f. sp. cubense tropical race 4 via antifungal metabolites and host defense induction
Source: Front Plant Sci. 2026 Aug 19;17:1863092. doi: 10.3389/fpls.2026.1863092 (PMC13534054; doi:10.3389/fpls.2026.1863092)
Supplement: Supplementary file 1 [file DataSheet1.doc]

**Supplementary Figs legends**

**Fig. S1** The inhibitory effects of different extract concentrations on the growth of Foc TR4 on PDA medium. Values are mean of three independent replicates ± SE. Different lowercase letters indicate significant differences (*p* ≤ 0.05).

**Fig. S2** Analysis of enzyme classification for strain 5-6 based on the CAZy database. AA: Auxiliary Activities. CBM: Carbohydrate-Binding Modules. CE: Carbohydrate Esterases. GH: Glycoside Hydrolases. GT: Glycosyl Transferases. PL: Polysaccharide Lyases.

**Fig. S1** The inhibitory effects of different extract concentrations on the growth of Foc TR4 on PDA medium. Values are mean of three independent replicates ± SE. Different lowercase letters indicate significant differences (*p* ≤ 0.05).

**Fig. S2** Analysis of enzyme classification for strain 5-6 based on the CAZy database. AA: Auxiliary Activities. CBM: Carbohydrate-Binding Modules. CE: Carbohydrate Esterases. GH: Glycoside Hydrolases. GT: Glycosyl Transferases. PL: Polysaccharide Lyases.
